# Case Report: Vertebro-vertebral arteriovenous fistula showing symptoms mimicking ALS: Diagnostic imaging supports accurate differentiation between ALS and mimicking conditions

**DOI:** 10.12688/f1000research.121554.1

**Published:** 2022-05-19

**Authors:** Hinako Kirikae, Ryuhei Harada, Tatsuhiko Hosaka, Tatsuro Misu, Daisuke Ando, Hitoshi Warita, Toshiki Endo, Shinya Sonobe, Kuniyasu Niizuma, Masashi Aoki

**Affiliations:** 1 School of Medicine, Tohoku University, Sendai, Miyagi, 980-8575, Japan; 2 Department of Neurology, Tohoku University Graduate School of Medicine, Sendai, Miyagi, 980-8574, Japan; 3 Department of Neurosurgery, Tohoku University Graduate School of Medicine, Sendai, Miyagi, 980-8574, Japan; 4 Department of Neurosurgical Engineering and Translational Neuroscience, Tohoku University Graduate School of Medicine, Sendai, Miyagi, 980-8575, Japan; 5 Department of Neurosurgical Engineering and Translational Neuroscience, Graduate School of Biomedical Engineering, Tohoku University, Sendai, Miyagi, 980-8575, Japan

**Keywords:** vertebro-vertebral arteriovenous fistula, amyotrophic lateral sclerosis, ALS mimics, angiography, diagnostic imaging

## Abstract

We report a rare case of a vertebro-vertebral arteriovenous fistula (VVAVF) manifesting as amyotrophic lateral sclerosis (ALS). A 76-year-old female patient presented with progressive weakness, muscle atrophy, fasciculation, and preserved deep tendon reflexes in the right upper limb. Electrophysiological testing showed lower motor neuron dysfunction. The patient was suspected to have ALS, but cervical magnetic resonance imaging (MRI) revealed enlarged blood vessels in the spinal canal, which compressed the cervical spinal cord and nerve roots. Angiography showed a shunt from the right vertebral artery to the right intervertebral vein and the vertebral venous plexus; therefore, the patient was diagnosed with VVAVF. Transarterial embolization was performed to obliterate the shunt, and weakness in the patient’s right upper limb subsequently improved. It is worth considering VVAVF as a differential diagnosis of ALS-like diseases.

## Introduction

Amyotrophic lateral sclerosis (ALS) is a progressive motor neuron disease that affects upper motor neurons (UMNs) and lower motor neurons (LMNs), causing muscle weakness and atrophy and, eventually, death. ^1^ Published criteria for diagnosing ALS include the assessment of clinical or electrophysiological signs of UMN and LMN dysfunction, as well as the exclusion of ALS-mimicking diseases. ^2^

Vertebro-vertebral arteriovenous fistula (VVAVF) is a rare vascular malformation defined as a direct shunt from the vertebral artery (VA) to the surrounding venous plexus. ^3^ Although the most frequent symptom of this disorder is bruit, some patients present with neurological symptoms such as muscle weakness, numbness, and pain. ^3^^,^^4^ VVAVF diagnosis is made via magnetic resonance angiography (MRA), computed tomography angiography (CTA) and/or digital subtraction angiography. ^5^ In many cases, subsequent endovascular or surgical treatment results in symptom improvement. ^3^^,^^5^

Here we describe a case of a patient with VVAVF presenting symptoms mimicking ALS.

## Case report

A 76-year-old unemployed Japanese woman presented to our hospital with a complaint of weakness in the right upper limb that had developed progressively over a period of more than eight months. The patient had a history of schizophrenia with onset at the age of 19, which included seizures and insomnia, and she was treated with oral medications of clotiazepam (20 mg three times a day every day), quetiapine (320 mg four times a day every day), sertraline (25 mg once a day every day), levetiracetam (500 mg twice a day every day), brotizolam (0.25 mg once a day every day), and suvorexant (15 mg once a day every day). She had no history of trauma or surgery. Her mother suffered from a mental illness of unknown details, and there is no other apparent family history. Neurological examination revealed moderate muscle atrophy and weakness in the right upper limbs with preserved reflexes, and fasciculation in the right first dorsal interosseous muscle.

Electromyography showed active and chronic denervation in the right biceps brachii and first dorsal interosseous muscle. Nerve-conduction studies showed repeater F-waves and decreased F-wave persistence in the bilateral median and ulnar nerves. These results suggested the presence of LMN dysfunction in the cervical region. Therefore, the patient appeared to meet the criteria for “possible ALS” according to the revised El Escorial criteria. ^6^

**Figure 1.**
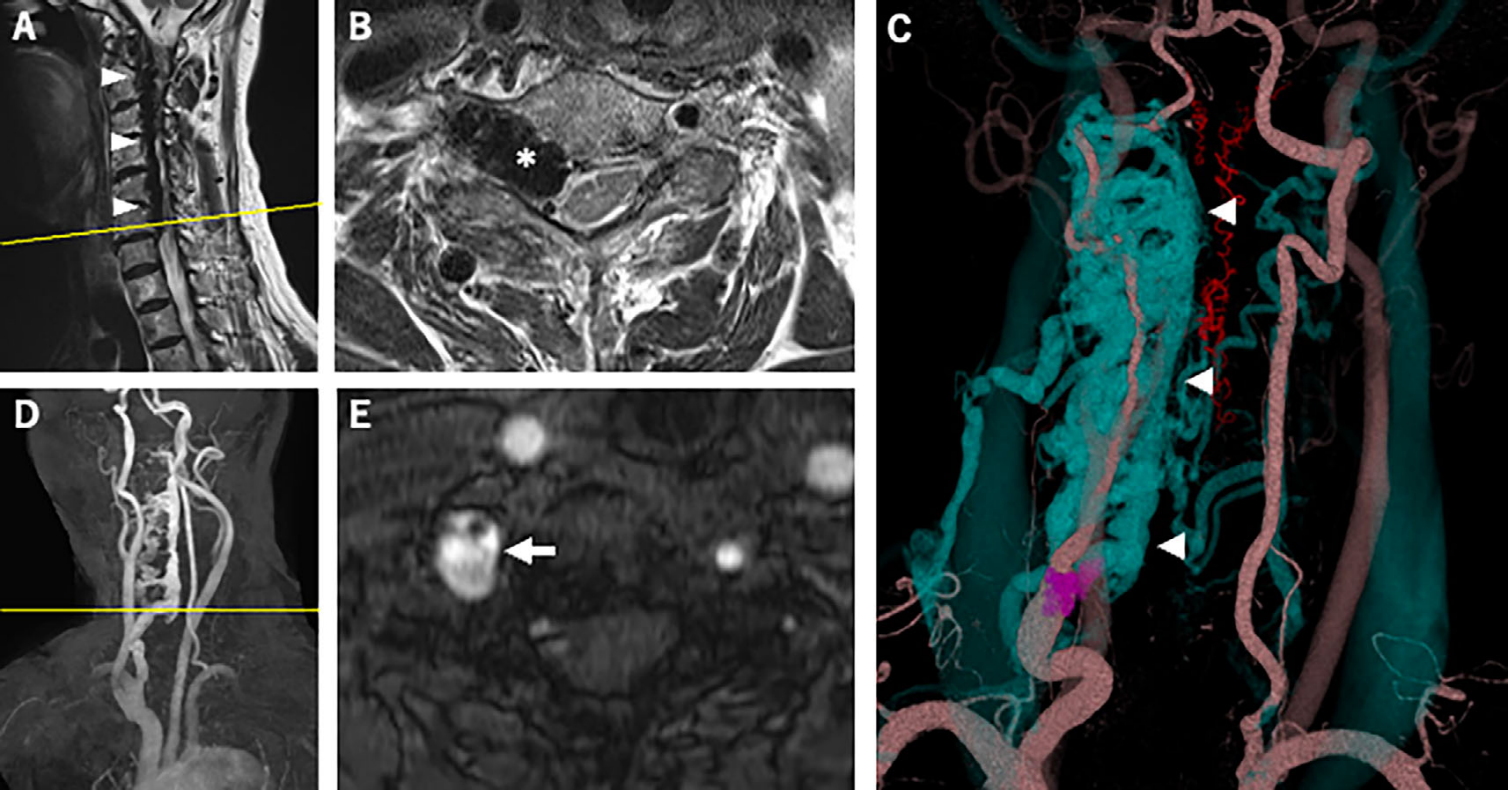
Magnetic resonance imaging and computed tomography angiography. (A) Sagittal T2-weighted imaging of the cervical spine showed the dilated internal vertebral venous plexus in the right side of the spinal canal from the C1 to C6/7 levels (arrowheads). (B) (the cross-section image of A at the yellow line) Axial T2-weighted imaging of the cervical spine showed the dilated right intervertebral vein (asterisk) at the C6/7 level compressing the spinal cord, and high signal intensity around the right AH at the same level. (C) Three-dimensional reconstruction of contrast-enhanced computed tomography revealed a dilated intervertebral vein and anterior internal vertebral venous plexus (blue, arrowheads). Arteriovenous fistula at the C7 level (purple) was also shown. (D) Oblique view of maximum-intensity projection cervical MRA showed a dilated right intervertebral vein and anterior internal vertebral venous plexus. The yellow line indicates the cross-sectional position of (E). (E) Axial image of time-of-flight cervical MRA showed an arteriovenous fistula at the C7 level (arrow). AH, anterior horn; MRA, magnetic resonance angiography.

**Figure 2.**
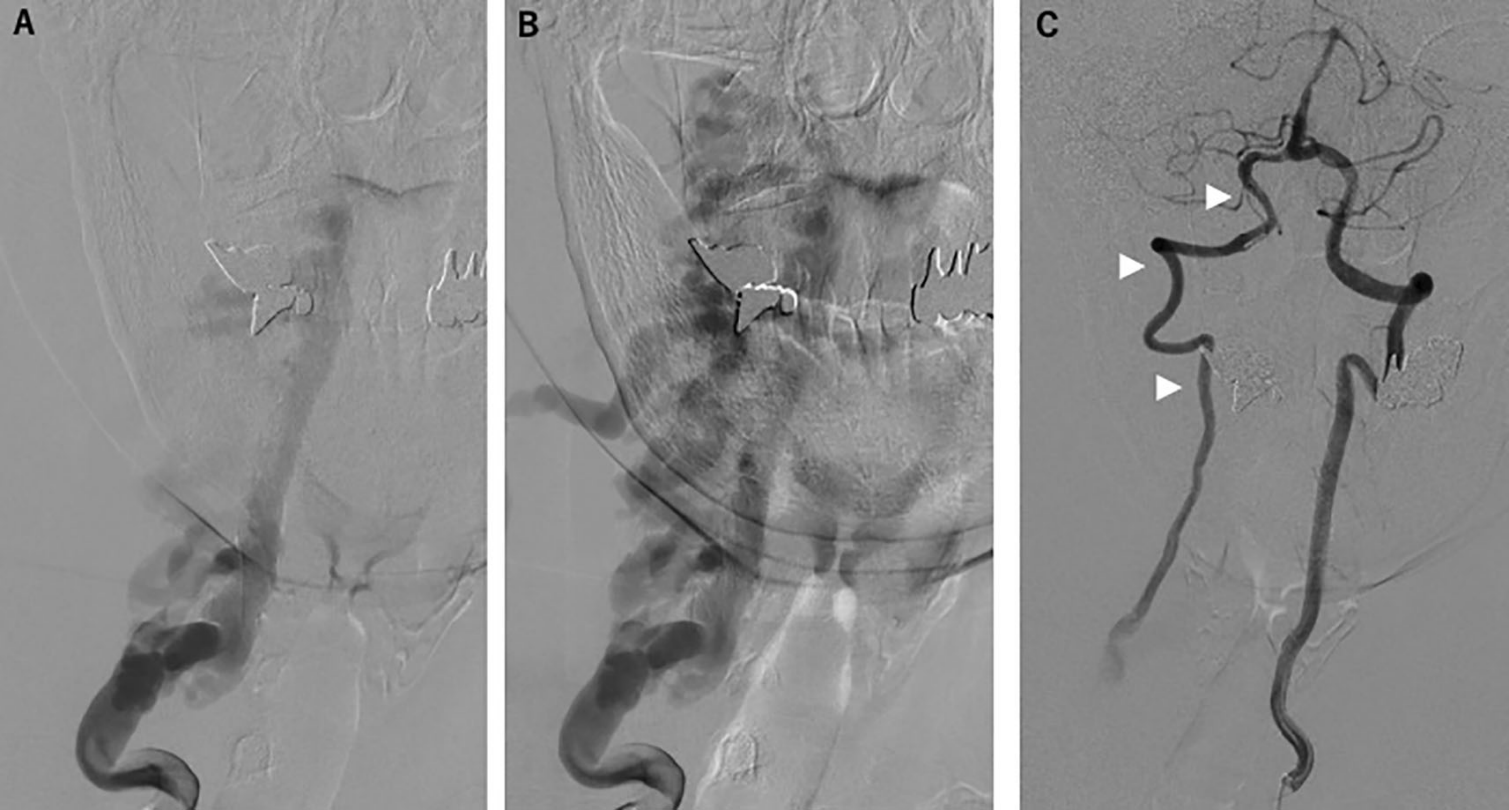
Preprocedural angiography. (A, B) Right vertebral angiography revealed the right vertebro-vertebral arteriovenous fistula with reflux to the anterior internal vertebral venous plexus via the right intervertebral vein. (C) Left vertebral angiography showed retrograde filling of the right vertebral artery distal to the arteriovenous fistula and running into the draining vein (arrowheads).

However, cervical magnetic resonance imaging (MRI) demonstrated a dilated internal vertebral venous plexus in the right side of the spinal canal from the C1 to C6/7 levels ( Figure 1A); this imaging showed compression of the spinal cord and spinal nerve roots at the same level, most severely at the C6/7 level ( Figure 1B). Axial T2-weighted imaging showed high signal intensity around the right anterior horn (AH) at that level ( Figure 1B). MRA and CTA revealed an arteriovenous fistula from the right VA to the right vertebral vein at the C6/7 level and showed dilation of the right intervertebral vein and the vertebral venous plexus ( Figure 1C- E). Right vertebral angiography revealed right VVAVF with reflux to the anterior internal vertebral venous plexus via the right intervertebral vein ( Figure 2A and B). Left vertebral angiography showed retrograde filling of the right VA distal to the arteriovenous fistula and running into the draining vein ( Figure 2C).

**Figure 3.**
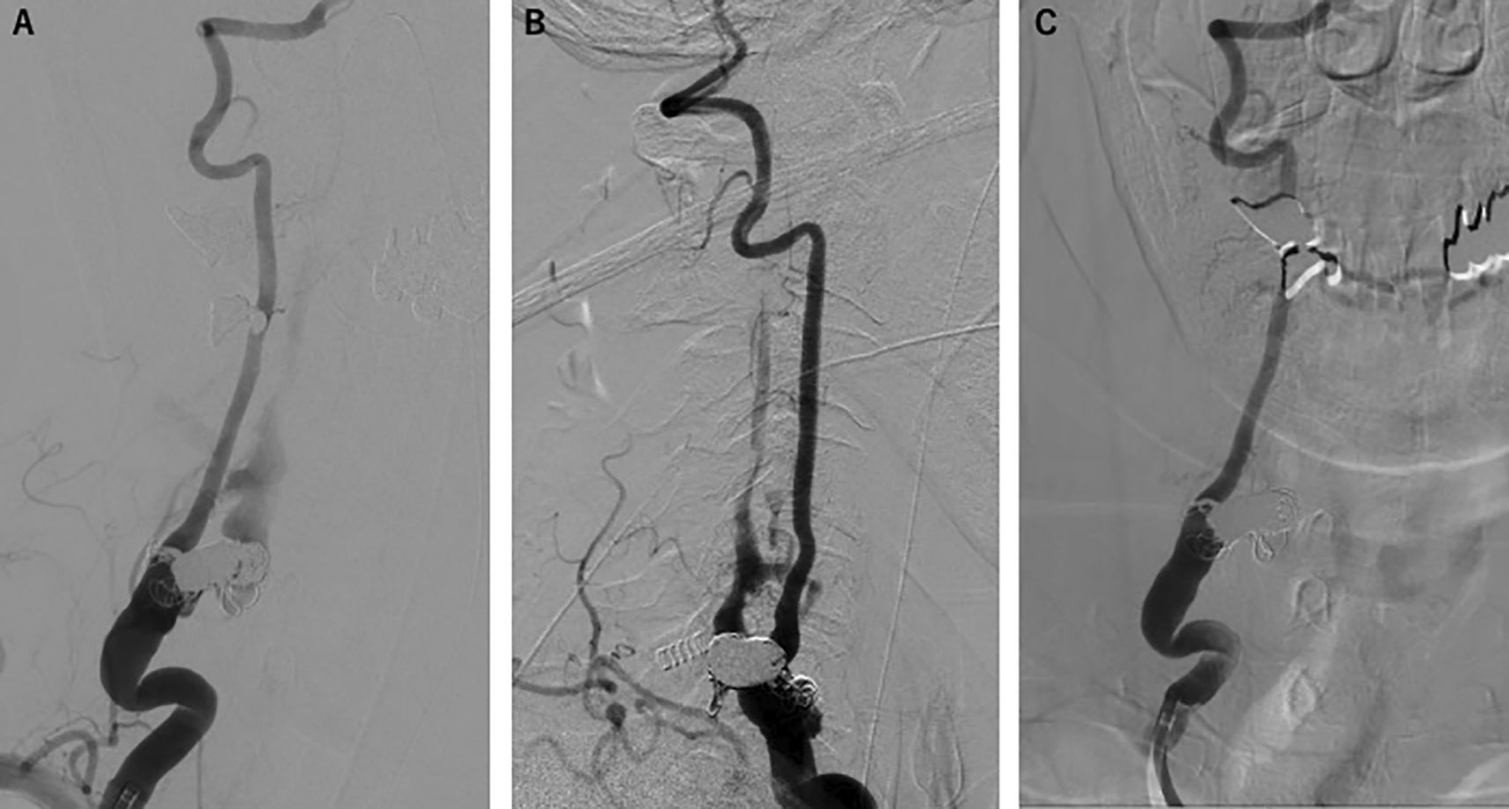
Postprocedural angiography. (A) Anteroposterior and (B) lateral right vertebral angiography showed remarkable reduction of the shunt flow. (C) Three months post-fistula embolization, anteroposterior right vertebral angiography demonstrated complete disappearance of the shunt flow.

Transarterial embolization of the fistula was performed using coil and n-butyl cyanoacrylate with no adverse events. There were no significant problems with intervention adherence and tolerability. Postprocedural angiography demonstrated a significant reduction of the shunt blood flow ( Figure 3A and B). Three months after treatment, a follow-up angiography was performed and revealed complete disappearance of the shunt blood flow ( Figure 3C). Furthermore, the presenting weakness in the patient’s right upper proximal limb slightly improved without the occurrence of any new neurological abnormalities.

## Discussion

VVAVF is a rare vascular malformation, and, to our knowledge, a total of 293 cases have been reported from 1990 to 2018, of which 280 cases were well-documented. Of these 280 cases and the present case, 136 cases (49%) were spontaneous, 76 (27%) were traumatic, and 68 (24%) were iatrogenic. ^3^ The male/female ratios of spontaneous, traumatic, and iatrogenic VVAVF were 1:2, 2:1 and 1:1, respectively. ^3^ Although the etiology of VVAVF was not identified in the present case, spontaneous VVAVF would be associated with connective tissue disorders including Ehlers-Danlos syndrome, neurofibromatosis type 1, and fibromuscular dysplasia. ^3^ Among the variety of presenting symptoms and clinical observations in VVAVF, bruit is the most common clinical manifestation. ^3^ Other frequently observed symptoms include weakness and numbness, pain, and headache. ^3^ On fewer occasions, tinnitus, subarachnoid hemorrhage, and congestive heart failure have also been reported. ^3^^,^^4^ Radiculopathy has been rarely reported as a manifestation of VVAVF. ^4^

Symptoms in the present case were likely caused by compression of the spinal cord and the ventral nerve root, as the patient’s symptoms were localized to the right upper limb without sensory involvement and partially improved following shunt-vessel embolization. The dilated blood vessels compressed the spinal cord from the C1 to C7 levels—most severely at the C6/7 level—and a high signal intensity around the right AH at that level was revealed via T2-weighted imaging, whereas no signal change was observed in the spinal cord at other affected levels. This suggests that the AH cells at the C6/7 level were more severely affected than in the other regions, leading to the patient’s partial posttreatment improvement. Furthermore, it is likely that the AH and the ventral nerve root were selectively damaged by spinal-cord compression, resulting in ALS-like symptoms brought on by damage to the affected UMNs and LMNs.

In the present case, the patient’s initial presenting symptoms were progressive UMN and LMN signs in the right upper limb, which are classified by current diagnostic criteria as possible ALS. According to criteria for diagnosis of ALS—including revised El Escorial, Awaji, and recently proposed Gold Coast criteria—the clinical or electrophysiological signs of UMN and LMN dysfunction, and exclusion of other possible diagnoses, are necessary for accurate ALS diagnosis. ^2^^,^^6^^,^^7^

Certain medical conditions that can be described as “ALS mimics” show motor neuron dysfunctions similar to those of ALS. ALS mimics tend to present atypical initial symptoms, namely the absence of LMN signs via electromyography and the absence of isolated UMN or LMN signs in the physical examination. ^8^ Other atypical signs suggesting ALS mimics include patient age younger than 50 years, slow or no progression of symptoms, and involvement of sensory symptoms. ^9^ A recent retrospective study conducted at an ALS clinic in Argentina found that 11.7% of patients with motor neuron disease symptomatology were later diagnosed as having ALS mimics. ^8^ Furthermore, even after being diagnosed as ALS, fully 3.9–9.7% of cases later turned out to be ALS mimics. ^8^^,^^9^ The alterative diagnoses of those patients include spinal cord pathology, hereditary spastic paraparesis, neuropathy, inclusion body myositis, multiple sclerosis, and paraneoplastic syndrome. ^8^^–^^10^ Accurate diagnosis of ALS mimics—even following an initial incorrect diagnosis of ALS—can lead to improved treatment options and higher quality of life.

Vascular malformations including epidural AVF, dural AVF, and perimedullary AVF may present symptoms mimicking ALS ^11^^,^^12^ and may lead to spinal-cord compression that can be treated by endovascular or surgical treatment. ^4^^,^^11^^,^^12^ To our knowledge, this is the first case report of VVAVF with symptoms mimicking ALS.

When diagnosing a patient with an ALS-like clinical presentation, it is critical to consider all the other possible diagnoses. More precisely, diagnostic imaging such as computerized tomography (CT), CTA, MRI, and/or MRA should be performed to differentiate from alternative diagnoses, including VVAVF.

## Conclusions

We report a case of VVAVF presenting motor neuron symptoms mimicking ALS. VVAVF is treatable and is worth considering as a differential diagnosis despite its clinical rarity, and diagnostic imaging should be performed when a patient presents with motor neuron dysfunctions resembling those of ALS.

## Data availability

### Underlying data

All data underlying the results are available as part of the article and no additional source data are required.

## Consent

Written informed consent for publication of their clinical details and clinical images was obtained from the patient.
