# Supplementary material for: Case Report: Vertebro-vertebral arteriovenous fistula showing symptoms mimicking ALS: Diagnostic imaging supports accurate differentiation between ALS and mimicking conditions
Source: F1000Res. 2022 Oct 13;11:546. Originally published 2022 May 19. [Version 3] doi: 10.12688/f1000research.121554.3 (PMC11401983; doi:10.12688/f1000research.121554.3)
Supplement: Peer review history

# Reviewer report: Reviewer response for version 3

**DOI:** 10.5256/f1000research.139731.r262993

**Published:** 2024-09-14

**Authors:** Matias Costa

**Affiliations:** 1 Swedish Medical Center, Seattle, USA

This case report discusses a rare vascular malformation called VVAVF, with symptoms mimicking ALS (Amyotrophic Lateral Sclerosis). VVAVF can be spontaneous, traumatic, or iatrogenic, with various symptoms including bruit, weakness, numbness, and headache. The case shown presented progressive upper and lower motor neuron signs, initially resembling ALS. However, diagnostic imaging revealed spinal cord compression due to VVAVF, suggesting an ALS mimic. Accurate diagnosis of ALS mimics is crucial for appropriate treatment and improved quality of life. VVAVF, though rare, should be considered in patients with ALS-like symptoms, emphasizing the importance of diagnostic imaging for differential diagnosis.

 This is a relevant paper for the practitioner that finds severe myelopathic signs / bulbar in a patient with otherwise no medical history or very young like in this case.

 I consider this paper relevant, and can be approved for indexing as is.

 The reference provided has a case in which a patient had similar MRI findings with spinal cord signal and prominent venous engorgement inside the canal. {Matias Costa et al. (2023 [Ref - 1])}

Are enough details provided of any physical examination and diagnostic tests, treatment given and outcomes?

Yes

Is the case presented with sufficient detail to be useful for other practitioners?

Yes

Is sufficient discussion included of the importance of the findings and their relevance to future understanding of disease processes, diagnosis or treatment?

Yes

Is the background of the case’s history and progression described in sufficient detail?

Yes

Reviewer Expertise:

Neurosurgery / Endovascular / Cerebrovascular

I confirm that I have read this submission and believe that I have an appropriate level of expertise to confirm that it is of an acceptable scientific standard.

## References

[1] : Vertebral-Venous fistulas: Single center experience and practical treatment approach. *Interv Neuroradiol* .2023; 10.1177/15910199231170079 15910199231170079 10.1177/15910199231170079 37073124 PMC12280272

# Reviewer report: Reviewer response for version 3

**DOI:** 10.5256/f1000research.139731.r153200

**Published:** 2022-11-17

**Authors:** Corrado Angelini

**Affiliations:** 1 Neuromuscular Laboratory, Department of Neurosciences, University of Padova, Padua, Italy

The present version appears improved and contains more detailed neurophysiology lab exams.

Are enough details provided of any physical examination and diagnostic tests, treatment given and outcomes?

Yes

Is the case presented with sufficient detail to be useful for other practitioners?

No

Is sufficient discussion included of the importance of the findings and their relevance to future understanding of disease processes, diagnosis or treatment?

No

Is the background of the case’s history and progression described in sufficient detail?

Partly

Reviewer Expertise:

Neuromuscular Disorders.

I confirm that I have read this submission and believe that I have an appropriate level of expertise to confirm that it is of an acceptable scientific standard.

# Reviewer report: Reviewer response for version 2

**DOI:** 10.5256/f1000research.136668.r148368

**Published:** 2022-09-30

**Authors:** Corrado Angelini

**Affiliations:** 1 Neuromuscular Laboratory, Department of Neurosciences, University of Padova, Padua, Italy

This is a possible treatable cause of mimicking ALS syndrome, the case might be susceptible to surgical treatment, so recognition of this entity is important. In this case description timing of muscle atrophy recovery is not described. Was sensory deficit investigated by sensory nerve conduction, SSEP (Somatosensory Evoked Potentials), or neurophysiology? In some motor neuron disease cases, there might be an abnormality of sensory function also in ALS (Facco E.et al.1989), however different from the described case, a differential statement might be useful.

Are enough details provided of any physical examination and diagnostic tests, treatment given and outcomes?

Yes

Is the case presented with sufficient detail to be useful for other practitioners?

No

Is sufficient discussion included of the importance of the findings and their relevance to future understanding of disease processes, diagnosis or treatment?

No

Is the background of the case’s history and progression described in sufficient detail?

Partly

Reviewer Expertise:

Neuromuscular Disorders.

I confirm that I have read this submission and believe that I have an appropriate level of expertise to confirm that it is of an acceptable scientific standard, however I have significant reservations, as outlined above.

## References

[1] : Sensory-motor conduction time in amyotrophic lateral sclerosis. *Riv Neurol* .59(3) :108-12 2595209

# Response

**Published:** 2022-10-11

**Authors:** Ryuhei Harada

**Affiliations:** Tohoku University Hospital, Japan

Dear Dr. Corrado Angelini,

 Thank you very much for the thoughtful and constructive feedback. We are thankful for the time and energy you expended.

 Our responses to your comments are as follow:

 > *"This is a possible treatable cause of mimicking ALS syndrome, the case might be susceptible to surgical treatment, so recognition of this entity is important."*

**Response:** Thank you very much for your comment. We agree that it is important to recognize the other treatable diseases when diagnosing a patient with an ALS-like clinical presentation.

**Q1:** *"In this case description timing of muscle atrophy recovery is not described."*

**A1：**Thank you for this suggestion.

 Muscle atrophy of the patient's right upper proximal limb remained after treatment, but weakness slightly improved. We have reflected this comment in lines 76-77 from the top of the “Case report” section.

**Q2:** *"Was sensory deficit investigated by sensory nerve conduction, SSEP (Somatosensory Evoked Potentials), or neurophysiology? In some motor neuron disease cases, there might be an abnormality of sensory function also in ALS (Facco E.et al.1989), however different from the described case, a differential statement might be useful."*

**A2：**Thank you for raising this point, and for the reference.

 Sensory nerve conduction studies revealed a mild decrease of the amplitude in bilateral ulnar nerves (8 μV for the left ulnar nerve, 11 μV for the right ulnar nerve), which were otherwise normal.

 Right upper extremity SSEP revealed a decrease in the amplitude of N9, which could be the effect of the compression of the spinal nerve roots at C1 to C6/7 level by dilated blood vessels, while central conduction time was normal. Left upper extremity and bilateral lower extremities SSEPs showed no abnormalities. We have reflected this comment in lines 18-20 and 32-34 from the top of the “Case report” section.

 Again, thank you for allowing us to strengthen our manuscript with your valuable comments and queries. We have worked hard to incorporate your feedback and hope that these revisions persuade you to accept our submission.

 Sincerely,

 Ryuhei Harada

 Department of Neurology, Tohoku University Graduate School of Medicine

# Reviewer report: Reviewer response for version 1

**DOI:** 10.5256/f1000research.133427.r142017

**Published:** 2022-07-12

**Authors:** Masafumi Hiramatsu

**Affiliations:** 1 Department of Neurological Surgery, Graduate School of Medicine, Dentistry and Pharmaceutical Sciences, Okayama University, Okayama, Japan

The authors report a case of VVAVF (Vertebro-vertebral arteriovenous fistula) with ALS-like symptoms. The present case is a typical case of VVAVF and the authors suggest that novelty exists only because ALS was required for differential diagnosis.

 When there are few abnormal findings in imaging studies, it may be challenging to differentiate the case from ALS. On the other hand, in the present case, the abnormalities are evident in imaging studies, and it is questionable whether ALS should be listed as a differential diagnosis. In the setting of progressive paralysis and muscle atrophy in the upper extremities, imaging studies of cervical spine lesions are often performed before suspecting ALS.

- The presence or absence of trauma or findings suspicious of connective tissue disorder should be noted.
- The presence or absence of bruit or findings suspicious of radiculopathy, such as pain and numbness, should be noted.
- The authors should state whether the findings described in the case report are upper or lower motor neuron dysfunction.
- The CTA shows red coiling vessels suggesting intradural reflux of shunt flow. Is there reflux on angiography? Intradural reflux may cause progressive congestive myelopathy. How about the presence of myelopathy on MR imaging and clinical examination?
- Right vertebral angiography shows the steal phenomenon, but is there any symptom of stealing, such as vertebra-basilar ischemia?
- On treatment, the coil mass appears to protrude into the VA; if the coil protrudes into the VA, there is a risk of thrombosis and stroke. It would be better to reconstruct the VA with a stent or occlude the VA completely.
- What do the authors believe is causing the hyperintensity signal in the anterior horn at the C6/7 level, spinal cord compression, or myelopathy due to intradural reflux of abnormal shunt flow?
- The authors state that the damaged anterior horn leads to partial posttreatment improvement, but I think the mass effect of the coil is the cause of the limitation of the symptom improvement.

Are enough details provided of any physical examination and diagnostic tests, treatment given and outcomes?

Partly

Is the case presented with sufficient detail to be useful for other practitioners?

Partly

Is sufficient discussion included of the importance of the findings and their relevance to future understanding of disease processes, diagnosis or treatment?

Partly

Is the background of the case’s history and progression described in sufficient detail?

Yes

Reviewer Expertise:

"dural arteriovenous fistula", "spinal arteriovenous fistula", "neuroendovascular therapy"

I confirm that I have read this submission and believe that I have an appropriate level of expertise to state that I do not consider it to be of an acceptable scientific standard, for reasons outlined above.

# Response

**Published:** 2022-07-29

**Authors:** Ryuhei Harada

**Affiliations:** Tohoku University Hospital, Japan

Dear Dr. Hiramatsu,

 Thank you very much for the thoughtful and constructive feedback. We are thankful for the time and energy you expended.

 Our responses to your comments are as follow:

**Q1:** " *The authors report a case of VVAVF (Vertebro-vertebral arteriovenous fistula) with ALS-like symptoms. The present case is a typical case of VVAVF and the authors suggest that novelty exists only because ALS was required for differential diagnosis.*

*When there are few abnormal findings in imaging studies, it may be challenging to differentiate the case from ALS. On the other hand, in the present case, the abnormalities are evident in imaging studies, and it is questionable whether ALS should be listed as a differential diagnosis. In the setting of progressive paralysis and muscle atrophy in the upper extremities, imaging studies of cervical spine lesions are often performed before suspecting ALS."*

**A1：**Thank you for raising an important point. We agree that the diagnosis of VVAVF was clear from the imaging findings. However, in the progressive course, upper motor neuron signs and lower motor neuron signs were observed, especially muscle atrophy, weakness, and fasciculation, and the clinical findings were mimicking ALS. On the other hand, VVAVF is rare, and the present patient had no past medical history that may be involved in the development of VVAVF (more detailed description in Q2). From this point of view, we considered it to be an instructive case.

**Q2:** *"The presence or absence of trauma or findings suspicious of connective tissue disorder should be noted."*

**A2：**Thank you for this suggestion.

 The patient had no history of trauma other than a history of a fracture of the left upper arm at the age of 68. The patient had no findings suggesting connective tissue diseases (e.g., tissue fragility, hyperextensibility of skin or joints, neurofibroma, elevated inflammatory markers, or collagen disease-related autoantibodies). We have reflected this comment by lines 8-11 from the top of the “Case report” section.

**Q3: *"*** *The presence or absence of bruit or findings suspicious of radiculopathy, such as pain and numbness, should be noted."*

**A3：**Thank you for this suggestion.

 After reviewing the imaging findings, a careful review of the physical examination revealed a bruit in the right neck and a slight tingling sensation in the radial side of the right forearm. Symptoms of pain were not present. We have reflected this comment by lines 29-31 from the top of the “Case report” section.

**Q4: *"*** *The authors should state whether the findings described in the case report are upper or lower motor neuron dysfunction."*

**A4：**Thank you for this suggestion.

 Preserved reflexes in the right upper extremity with muscle atrophy reflect upper motor neuron dysfunction. Muscle atrophy and fasciculation in the right upper extremity reflect lower motor neuron dysfunction. We have reflected this comment by lines 13-15 from the top of the “Case report” section.

**Q5:** "The CTA shows red coiling vessels suggesting intradural reflux of shunt flow. Is there reflux on angiography? Intradural reflux may cause progressive congestive myelopathy. How about the presence of myelopathy on MR imaging and clinical examination?"

**A5：**Thank you for providing these discussions.

 The red coiling vessels of figure 1C represent anterior and posterior spinal arteries.

 Angiography did not reveal reflux into them, and their involvement in the arteriovenous shunt was not apparent. We have not observed clinical or imaging findings suggestive of congestive myelopathy.

 We have reflected this comment by figure legend 1C and lines 36-37 from the top of the “Case report” section.

**Q6:** *"Right vertebral angiography shows the steal phenomenon, but is there any symptom of stealing, such as vertebra-basilar ischemia?"*

**A6：**Thank you for this suggestion. The patient had no symptoms suggestive of vertebra-basilar ischemia (e.g., vertigo, dizziness, loss of vision, nausea, vomiting, and dysarthria). We have reflected this comment by lines 34-36 from the top of the “Case report” section.

**Q7: *"*** *On treatment, the coil mass appears to protrude into the VA; if the coil protrudes into the VA, there is a risk of thrombosis and stroke. It would be better to reconstruct the VA with a stent or occlude the VA completely."*

**A7：**Thank you for the important advice.

 Follow-up angiography after 3 months of treatment showed residual protrusion of the coil mass into the VA, although the blood flow in the VA main trunk was maintained. Therefore, we agree that careful follow-up is needed.

**Q8:** *"What do the authors believe is causing the hyperintensity signal in the anterior horn at the C6/7 level, spinal cord compression, or myelopathy due to intradural reflux of abnormal shunt flow?"*

**A8：**Thank you for this suggestion. You have raised an important question.

 We consider the hyperintensity signal in the anterior horn at the C6/7 level to be caused primarily by spinal cord compression by dilated blood vessels.

 The level of intramedullary hyperintensity signal coincidences with the site of the most intense spinal cord compression. It is a finding that supports the mechanism of spinal cord compression. On the other hand, the intramedullary lesion appears too localized to be considered congestive myelopathy. As noted above, there is also no apparent reflux of the anterior and posterior spinal arteries, nor is there apparent regurgitation in the spinal canal.

 Please see lines 13-23 from the top of the “Discussion” section.

**Q9:** *"The authors state that the damaged anterior horn leads to partial posttreatment improvement, but I think the mass effect of the coil is the cause of the limitation of the symptom improvement."*

**A9: **Thank you for providing these insights.

 Indeed, I agree with the point that the mass effect of the coils may offset the improvement in spinal cord compression due to the reduction of dilated vessels. However, we have observed improvement in clinical symptoms and imaging findings of spinal cord compression after endovascular treatment.

 Again, thank you for giving us the opportunity to strengthen our manuscript with your valuable comments and queries. We have worked hard to incorporate your feedback and hope that these revisions persuade you to accept our submission.

 Sincerely,

 Ryuhei Harada

 Department of Neurology, Tohoku University Graduate School of Medicine
